# Stomatal development in the changing climate

**DOI:** 10.1242/dev.202681

**Published:** 2024-10-21

**Authors:** Li Cong Chua, On Sun Lau

**Affiliations:** Department of Biological Sciences, National University of Singapore, 14 Science Drive 4, Singapore 117557, Singapore

**Keywords:** Stomatal development, Environmental stress and signaling, Developmental plasticity, Climate change

## Abstract

Stomata, microscopic pores flanked by symmetrical guard cells, are vital regulators of gas exchange that link plant processes with environmental dynamics. The formation of stomata involves the multi-step progression of a specialized cell lineage. Remarkably, this process is heavily influenced by environmental factors, allowing plants to adjust stomatal production to local conditions. With global warming set to alter our climate at an unprecedented pace, understanding how environmental factors impact stomatal development and plant fitness is becoming increasingly important. In this Review, we focus on the effects of carbon dioxide, high temperature and drought – three environmental factors tightly linked to global warming – on stomatal development. We summarize the stomatal response of a variety of plant species and highlight the existence of species-specific adaptations. Using the model plant *Arabidopsis*, we also provide an update on the molecular mechanisms involved in mediating the plasticity of stomatal development. Finally, we explore how knowledge on stomatal development is being applied to generate crop varieties with optimized stomatal traits that enhance their resilience against climate change and maintain agricultural productivity.

## Teaser

**Summary:** This Review highlights the molecular mechanisms and species-specific adaptations in stomatal development in response to key stressors of climate change.

## Introduction

Stomata are microscopic pores flanked by a pair of guard cells (GCs; see Glossary, Box 1) on the surface of plants. Adjustments to GC turgor pressure allows the regulation of gas exchange (Nadeau and Sack, 2002). This enables plants to manage the intake of carbon dioxide (CO_2_) and the release of oxygen and water vapor, thereby balancing photosynthesis and water loss. In addition to being integral to plant physiology, stomata connect plant processes to the broader environmental and climatic contexts. When considering gas exchanges collectively at the global scale, stomata significantly contribute to the terrestrial carbon and water cycles (Hetherington and Woodward, 2003). These cycles are vital components of the global climate system, influencing weather patterns and climate regulation.

**Box 1. Glossary**

**Amplifying division.** Subsequent divisions of meristemoids to produce more stomatal precursor cells (i.e. meristemoids and SLGCs).

**Entry division.** The initial asymmetric cell division that occurs in a meristemoid mother cell (MMC, which is derived from a protodermal cell), signifying the start of stomatal development.

**Guard cell (GC).** Specialized cells flanking each stomatal pore, regulating its opening and closing to control gas exchange (CO_2_ uptake and O_2_ release) and water loss. Changes in turgor pressure within the guard cells determine the stomatal aperture.

**Meristemoid mother cell (MMC).** The initial cell in the stomatal lineage that divides asymmetrically to produce a meristemoid and an SLGC.

**One-cell-spacing rule.** A principle of stomatal patterning whereby stomata are separated by at least one non-stomatal epidermal cell, ensuring efficient function of GCs for optimal gas exchange.

**Spacing division.** Asymmetric divisions of SLGCs that have adopted the MMC fate. These divisions generate a secondary meristemoid positioned away from existing stomata/stomatal precursor cells such that they are spaced by at least one non-stomatal cell.

**Stomatal conductance.** A measure of the rate at which CO_2_ enters, or water vapor exits, through the stomata, reflecting the efficiency of gas exchange and transpiration. It is influenced by stomatal density, size and opening.

**Stomatal density (SD).** Measured as the number of stomata per unit area, it provides insight into how densely stomata are packed, which is key for assessing a plant's capacity for gas exchange.

**Stomatal index (SI).** The ratio of stomata to the total number of epidermal cells (including stomata) within a specific area, offering a normalized view of stomatal frequency relative to overall cell count.

**Subsidiary cell (SC).** Specialized cells adjacent to guard cells in some plant species that assist in stomatal opening and closing by providing structural support and facilitating the movement of GCs. Typically found in grasses, they help regulate the aperture of the stomatal pore.

First identified in the fossil record about 400 million years ago, the basic form of stomata has remained largely unchanged compared with modern plants (Ravan, 2002). However, many plant species possess the ability to adjust the production of stomata in response to a wide range of environmental signals (Radin et al., 1994; Woodward and Kelly, 1995). These responses support plants to adapt to diverse habitats and have contributed to the evolutionary success of terrestrial plants over millennia (Beerling and Chaloner, 1993a). In spite of this, the rapid change in climate as a result of global warming is challenging plant growth and survival. Understanding how the changing climate influences stomatal response and production will be crucial for predicting and mitigating its impact on vegetation patterns and agricultural systems.

Current projections of global climate change indicate significant shifts in environmental conditions that could have detrimental effects on plant life and agriculture. Human activities have significantly disrupted the global carbon cycle, leading to a steady rise in atmospheric CO_2_ levels. Presently, atmospheric CO_2_ concentrations stand at about 415 ppm and are projected to increase to approximately 685 ppm by 2103, driven by net anthropogenic emissions that added an estimated 5.2 gigatons of CO_2_ to the atmosphere in 2022 alone (Friedlingstein et al., 2022; IPCC, 2023; Zheng and Chen, 2023). Additionally, temperature is projected to increase by 1.5-2°C within this century, which will exacerbate extreme climate events, such as intense droughts and erratic precipitations, potentially undermining agricultural productivity and ecosystem stability globally (IPCC, 2023). These changes will compel a shift in traditional farming practices and the adoption of new agricultural technologies to maintain productivity.

In this Review, we summarize our current understanding on how global warming-related stresses impact stomatal production in a variety of plant species and discuss the underlying molecular mechanisms, which were mostly revealed through the model plant *Arabidopsis*. We first introduce how stomata are generated in plants, describing the key regulators and signaling pathways involved. We then focus on three environmental factors that are closely associated with climate change: increase in CO_2_ levels, high temperatures and drought. We describe their impacts on stomatal development and the signaling pathways involved, and conclude with a discussion on the outstanding questions and challenges. Although our primary focus is on these key stressors, many other environmental factors, such as flooding, humidity fluctuations and ozone, also influence stomatal formation (Tricker et al., 2012; Yordanova et al., 2005; Elagoz et al., 2006).

## Overview of stomatal development and the core regulatory pathways

### Stomatal formation in eudicots and grasses

**Fig. 1.**
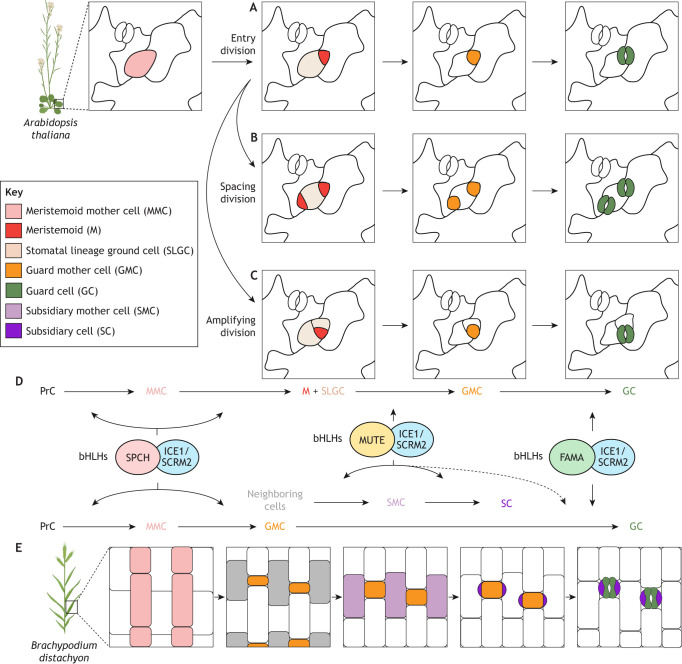
**Cell fate transitions during stomatal development in *Arabidopsis* and *Brachypodium*.** (A) In *Arabidopsis thaliana*, the stomatal cell lineage begins with the meristemoid mother cell (MMC; pink), which divides asymmetrically to produce a smaller meristemoid (M; red) and a larger stomatal lineage ground cell (SLGC; beige). The meristemoid matures into a guard mother cell (GMC; orange), which divides symmetrically to form kidney-shaped guard cells (GC; green). (B) The SLGC can re-adopt the MMC fate, generating another meristemoid away from the existing meristemoid through a spacing division, or differentiate into a pavement cell. (C) The meristemoid can also undergo self-renewing amplifying divisions to generate more stomatal lineage cells. (D) Stomatal lineage development is sequentially regulated by the related bHLH transcription factors SPCH, MUTE and FAMA, which form heterodimers with ICE1 (also known as SCRM) or SCRM2 to control downstream targets. In *Arabidopsis* (top pathway), SPCH-ICE1/SCRM2 drives the progression from protodermal cells (PrC) to MMC and M; MUTE-ICE1/ SCRM2 matures M cells to GMCs; and FAMA-ICE1/SCRM2 drives GMC division into GCs. In *Brachypodium* (bottom pathway), SPCH-ICE1/SCRM2 specifies PrCs to divide into GMCs; MUTE-ICE1/SCRM2 recruits lateral neighbor cells to adopt SMC fate and then divide into SCs; and FAMA-ICE1/SCRM2 drives GMC division into GCs. MUTE-ICE1/SCRM2 also aids in guiding proper division orientation during GMC division into GCs (dashed arrow), which is correlated with preventing GC complex abortion and is important for producing functional GCs. (E) In *Brachypodium distachyon*, stomata develop from base to tip and are specified within predetermined cell files. These cells undergo an initial asymmetric division to produce a smaller GMC and a larger inter-stomatal sister cell. Once the GMC identity is established, lateral cells from adjacent cell files (gray) take on the subsidiary mother cell (SMC; lilac) fate and divide asymmetrically, forming a pair of young subsidiary cells (SC; purple). The GMC then undergoes a symmetric division to produce dumbbell-shaped guard cells (GC; green), which mature alongside the young SCs, culminating in a mature four-celled stomatal complex. For illustration purposes, only stomatal and subsidiary cell lineages are highlighted. Protodermal cells (PrC), which can give rise to MMCs and other cell types, are not highlighted. This illustration was created with BioRender. bHLHs, basic helix-loop-helix transcription factors; ICE1, INDUCER OF CBF EXPRESSION 1; SCRM2, SCREAM 2; SPCH, SPEECHLESS.

In higher plants, stomatal GCs are the final differentiated cell stage of a specialized epidermal cell lineage called the stomatal lineage. In the dicot model *Arabidopsis thaliana*, the stomatal lineage begins when protodermal cells adopt the stomatal cell fate, transforming into meristemoid mother cells (MMCs; see Glossary, Box 1) (Fig. 1) (Nadeau and Sack, 2002; Lau and Bergmann, 2012; Han and Torii, 2016). Following an asymmetric entry division (see Glossary, Box 1), the MMC produces a smaller, triangular-shaped meristemoid and a larger stomatal lineage ground cell (SLGC) (Nadeau and Sack, 2002). The stem cell-like meristemoid undergoes either self-renewal through one to three rounds of amplifying divisions (see Glossary, Box 1) or differentiation into a guard mother cell (GMC), which eventually divides symmetrically to yield a pair of kidney-shaped GCs (Nadeau and Sack, 2002). Meanwhile, the SLGCs can either differentiate into pavement cells and exit the lineage, or adopt the MMC fate and undergo a spacing division (see Glossary, Box 1), generating a satellite meristemoid distal to the existing meristemoid or GMC (Geisler et al., 2000). Owing to the scattered initiation of stomatal precursors, stomata are dispersed on the leaf epidermis in dicot plants (Geisler and Sack, 2002).

By contrast, grasses, which are the most studied group within the monocots, exhibit linear stomatal patterning in parallel epidermal cell rows (Stebbins and Jain, 1960). Stomatal development in grasses starts at the base of the leaf and progresses upwards (i.e. following an acropetal pattern in which early stomatal lineage cells are at the base and mature stomata are found at the tip of a leaf) (Nunes et al., 2020). Cereal crops, such as wheat, rice and maize, produce stomatal complexes consisting of dumbbell-shaped GCs flanked by pairs of subsidiary cells (SCs; see Glossary, Box 1) (Fig. 1). Initially, MMCs are specified within predetermined cell files at the base of a leaf. An MMC then divides asymmetrically to form a meristemoid (entry division), which quickly establishes the GMC fate, and a larger inter-stomatal sister cell (Stebbins and Jain, 1960). Because meristemoids in grasses do not undergo amplifying divisions, they are often directly referred to as GMCs (Rudall et al., 2017; Hepworth et al., 2018). Young GMCs recruit lateral cells in the adjacent cell files to adopt the subsidiary mother cell (SMC) fate; these SMCs will divide asymmetrically to generate young SCs (Stebbins and Jain, 1960; Nunes et al., 2020). Subsequently, a GMC divides symmetrically to form a pair of GCs, whereas the young SCs mature and enlarge alongside the GCs, ultimately forming a mature four-celled stomatal complex (Hepworth et al., 2018).

### Master transcription factors in stomatal lineage progression

Extensive genetic studies in *Arabidopsis* have identified key molecular players orchestrating the meristemoid-GMC-GC cell fate transitions. These transitions are primarily driven by the sequential activation of the basic helix-loop-helix (bHLH) transcription factors SPEECHLESS (SPCH), MUTE and FAMA (Fig. 1) (Ohashi-Ito and Bergmann, 2006; MacAlister et al., 2007; Pillitteri et al., 2007; Kanaoka et al., 2008). By broadly regulating the transcriptome, SPCH specifies the MMC and meristemoid identities and promotes stomatal asymmetric divisions that yield additional meristemoids (MacAlister et al., 2007; Lau et al., 2014). Mutants of *SPCH* are seedling lethal as they lack stomatal precursors and hence stomata entirely, whereas the overexpression of SPCH, particularly its MAP kinase-insensitive version (discussed further in the following section), can cause extra cell divisions (MacAlister et al., 2007; Lampard et al., 2008; Pillitteri et al., 2007). During the transition to the GMC stage, MUTE terminates the SPCH-driven asymmetric division and establishes the GMC fate through transcriptional reprogramming (Pillitteri et al., 2007; Han et al., 2018). Importantly, it directly activates multiple cell cycle regulators, culminating in a tightly regulated surge of gene expression that orchestrates the singular symmetric division of the GMC (Han et al., 2018, 2022). As such, loss-of-function mutation in *MUTE* leads to an inward spiral of small cells as a result of repetitive amplifying divisions of meristemoids (Pillitteri et al., 2007). Following MUTE, FAMA is expressed from late GMC to GC maturity, to complete the GMC-to-GC differentiation (Ohashi-Ito and Bergmann, 2006). FAMA confers GC identity by directly upregulating genes essential for stomatal function, and it also maintains the terminal identity of GCs by preserving the epigenomic landscape (Matos et al., 2014; Lee et al., 2014; Negi et al., 2013; Liu et al., 2024). Loss-of-function *fama* mutants display elongated clusters of cells as a result of continued divisions of GMCs (Ohashi-Ito and Bergmann, 2006). As bHLH transcription factors, which bind DNA as dimers, proper SPCH, MUTE and FAMA function depends on their obligate heterodimeric bHLH partners, INDUCER OF CBF EXPRESSION 1 (ICE1; also known as SCREAM, SCRM) and SCREAM 2 (SCRM2) (Kanaoka et al., 2008). ICE1 and SCRM2 have partially overlapping roles in stomatal development, with ICE1 being required throughout the progression of the lineage (Kanaoka et al., 2008). Additive mutations in ICE1 and SCRM2 progressively mimic the mutant phenotypes of *fama*, *mute* and *spch* (Kanaoka et al., 2008). In grasses, orthologs of these transcription factors are present and also play a role in stomatal cell fate transitions (see Box 2 for details).

**Box 2. Stomatal bHLH transcription factors in *Brachypodium***

*Brachypodium distachyon*, a wheat relative, has emerged as a prominent model for studying stomatal development in grasses, exhibiting both parallels and divergences in the use of bHLH transcription factors compared with *Arabidopsis* (Raissig et al., 2016, 2017; McKown et al., 2023). For instance, although BdSPCH1 and BdSPCH2 in *Brachypodium* jointly regulate stomatal cell fate initiation and specification similarly to *Arabidopsis*, they primarily act as fate determinants rather than promoting amplifying divisions, because grasses such as *Brachypodium* lack a self-renewing phase in their stomatal development (Raissig et al., 2016). Interestingly, BdMUTE assumes a unique role in *Brachypodium* by directing the recruitment of SCs through its intrinsic mobility, migrating from GMCs to adjacent cells to establish SMC fate (Raissig et al., 2017). Recent findings indicate that BdMUTE also plays a cell-autonomous role in GMCs, ensuring the proper orientation of the division plane to form two symmetrical GCs (Spiegelhalder et al., 2024). Notably, *bdmute* mutants are still capable of producing functional stomata, albeit with defects, whereas GMCs of *mute* mutants in maize (*Zea mays*) and rice (*Oryza sativa*) typically arrest after misoriented divisions (Raissig et al., 2017; Wu et al., 2019; Liu et al., 2009; Wang et al., 2019). By contrast, the role of FAMA in GC differentiation is relatively conserved across dicots and grasses, though it appears to play only a minor role in cell division regulation in grasses, as *bdfama* and *osfama* mutants do not exhibit continued GMC divisions but show undifferentiated or swollen GCs (Wu et al., 2019; McKown et al., 2023). Additionally, FAMA in grasses may also contribute to SC differentiation: whereas *bdfama* mutants still produce SCs of wild-type appearance, expressing *BdFAMA* under the *BdMUTE* promoter in *bdmute* mutants can partially rescue the SC recruitment defects seen in these mutants (McKown et al., 2023). This rescue is possibly because BdFAMA and BdMUTE share a subset of heterodimerization partners, allowing them to substitute for each other, but only when expressed during the developmental stage typically occupied by the missing partner (McKown et al., 2023). Moreover, knockout mutants of *FAMA* in rice (*Oryza sativa*) show aberrant SC morphology, with occasional stomatal complexes having only a single SC (Wu et al., 2019). Finally, in contrast to the partially redundant role of ICE1 and SCRM2 as heterodimeric partners of SPCH, MUTE and FAMA in *Arabidopsis*, BdICE1 and BdSCRM2 function as true paralogs in *Brachypodium* (Raissig et al., 2016). BdICE1 is primarily responsible for establishing stomatal fate, as its single mutant completely lacks stomata, whereas BdSCRM2 is crucial for the differentiation of the stomatal complex, with its single mutant producing defective four-celled stomatal complexes (Raissig et al., 2016).

### A core regulatory pathway in stomatal formation

**Fig. 2.**
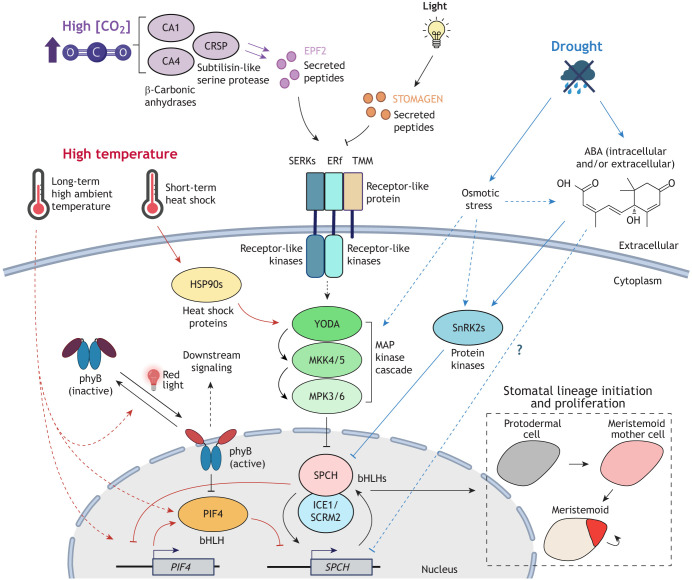
**Regulation of stomatal development by environmental signaling in *Arabidopsis*.** A mitogen-activated protein kinase (MAPK) cascade, led by the MAPKKK YODA, represses stomatal development in *Arabidopsis*. Upstream of the MAPK cascade is a ligand-receptor system consisting of the ERECTA family of receptor-like kinases, along with co-receptors SERKs and the receptor-like protein TMM, which bind to the EPIDERMAL PATTERNING FACTOR-LIKE (EPFL) family of secreted peptides, such as EPF2. In early stomatal lineage cells, activation of the MAPK cascade leads to the phosphorylation and degradation of the bHLH transcription factor SPCH to suppress stomatal lineage initiation and proliferation (dashed-line box). SPCH heterodimerizes with ICE1 or SCRM2, two redundant bHLH transcription factors, to bind and regulate the expression of downstream targets, including *SPCH*'s own expression, via a positive feedback loop. High CO_2_ levels activate the β-carbonic anhydrases CA1 and CA4 to promote the expression of the subtilisin-like protease *CRSP*, which cleaves the EPF2 propeptide into its active form. The mature EPF2 peptide binds and activates the ERf receptor complex, suppressing stomatal development. STOMAGEN, another EPFL family member, is light-responsive and competes with EPF2 for binding to the receptor complex, thereby inhibiting downstream signaling. Prolonged high ambient temperatures promote *PIF4* gene expression and protein stability, and PIF4 directly suppresses *SPCH* expression. SPCH also directly inhibits *PIF4* gene expression, resulting in a negative feedback loop. Elevated temperatures also convert photoreceptor phytochrome B (phyB) to its inactive form, relieving its inhibition on PIF4. The heat-induced inactivation of phyB may also suppress other branches of light signaling pathway that influence stomatal development. Under short-term heat shock, heat shock proteins (HSP90s) are activated and interact with YODA, activating the MAPK cascade to inhibit stomatal development. In drought conditions, SPCH proteins are destabilized to suppress stomatal formation. This may occur due to osmotic stress directly activating the MAPK pathway. Osmotic stress also elevates ABA levels and activates the SnRK2 protein kinases, which directly phosphorylate and suppress SPCH. Arrows indicate activations and T-bars indicate inhibitions. Solid lines represent confirmed biochemical interactions, and dashed lines represent indirect regulatory effects. Pathways triggered by CO_2_, high temperatures, and drought are highlighted with purple, red and blue arrows, respectively. This illustration was created using BioRender. ABA, abscisic acid; bHLHs, basic helix-loop-helix transcription factors; CAs, β-carbonic anhydrases; CRSP, CO_2_ RESPONSE SECRETED PROTEASE; EPF2, EPIDERMAL PATTERNING FACTOR 2; ERf, family of ERECTA; HSP90s, heat shock protein 90s; ICE1, INDUCER OF CBF EXPRESSION 1; MAP, mitogen-activated protein; MKK4/5, MITOGEN ACTIVATED PROTEIN KINASE KINASE 4/5; MPK3/6, MITOGEN ACTIVATED PROTEIN KINASE 3/6; phyB, phytochrome B; PIF4, PHYTOCHROME-INTERACTING FACTOR 4; SCRM2, SCREAM 2; SERKs, SOMATIC EMBRYOGENESIS RECEPTOR-LIKE KINASES; SnRK2s, SNF1-related protein kinase 2s; SPCH, SPEECHLESS; TMM, TOO MANY MOUTHS.

Upstream of the stomatal transcription factors, a conserved ligand-receptor system has been identified in regulating the formation and patterning of stomata. The system consists of the ERECTA family (ERf) of leucine-rich repeat receptor-like kinases (LRR-RLKs), their ligands from the EPIDERMAL PATTERNING FACTOR-LIKE (EPFL) family of secreted peptides (Shpak et al., 2005; Rychel et al., 2010) and additional co-receptors, including an LRR receptor-like protein, TOO MANY MOUTHS (TMM), and the LRR-RLK SOMATIC EMBRYOGENESIS RECEPTOR-LIKE KINASES (SERKs) (Fig. 2) (Nadeau and Sack, 2002; Lee et al., 2012; Meng et al., 2015; Yang and Sack, 1995). When activated by EPFL binding, the ERf-TMM-SERK receptor complex triggers the mitogen-activated protein kinase (MAPK) signaling cascade, which involves the MAPKKK YODA, the MAPKKs MKK4/5 and the MAPKs MPK3/6 (Bergmann et al., 2004; Wang et al., 2007; Lampard et al., 2009). In the early stomatal lineage cells of *Arabidopsis*, activation of the MAPK cascade leads to MPK3/6 targeting specific residues within the MAPK target domain (MPKTD) of SPCH, leading to the phosphorylation and degradation of SPCH (Lampard et al., 2008). Mutating these specific MPKTD residues can stabilize SPCH, resulting in excessive cell divisions (Lampard et al., 2008). Interestingly, ICE1 and SCRM2 scaffold the interaction between SPCH and MPK3/6, bringing SPCH into close proximity with MPK3/6 for its downregulation (Putarjunan et al., 2019). During this process, ICE1 and SCRM2 are also phosphorylated and degraded. The MPTKD is present on SPCH across angiosperms, suggesting a conserved MAPK-dependent regulatory mechanism for stomatal patterning through SPCH (Lampard et al., 2008; MacAlister and Bergmann, 2011). However, in *Brachypodium distachyon*, BdICE1, unlike AtICE1, contains high fidelity MAPK target sites and its overexpression, in contrast to BdSPCH1/2, does not lead to substantial cell divisions (Raissig et al., 2016). This suggests that the MAPK pathway in *Brachypodium* preferentially acts on BdICE1 and highlights species-specific differences in regulating stomatal development (MacAlister and Bergmann, 2011; Raissig et al., 2016).

An important role of this ligand-receptor system is restricting stomatal formation and maintaining proper spacing of GCs for efficient gas exchange, i.e. enforcing the ‘one-cell-spacing’ rule (see Glossary, Box 1) (Geisler et al., 2000; Dow et al., 2014b). For instance, EPF2, secreted by MMCs and early meristemoids, inhibits neighboring ERECTA-expressing cells from acquiring stomatal lineage fate through activation of the MAPK signaling pathway, and the pairing of EPF1 and ERECTA-LIKE 1 (ERL1) inhibits MUTE activity and prevents cells adjacent to existing stomatal lineage cells from developing into GCs (Qi et al., 2017; Lee et al., 2012; Hara et al., 2007; Hunt and Gray, 2009). However, although EPF1-ERL1 is genetically upstream of the MAPK cascade, MUTE lacks the predicted MPKTD, and there is no direct biochemical evidence of its downregulation through the cascade (Qi et al., 2017). This raises questions about whether MUTE is a direct substrate of the EPF1-ERL1-activated MAPK signaling cascade. Interestingly, STOMAGEN (another EPFL family member; also known as EPFL9) competes with EPF1/2 for binding to the ERf and thus positively regulates stomatal formation (Lee et al., 2015; Sugano et al., 2010). As illustrated below, the regulation of the EPFL family by environmental signals allows plants to modulate stomatal production (Fig. 2) (Engineer et al., 2014; Wang et al., 2021).

Importantly, this EPF peptide-regulated MAPK signaling is likely conserved in grasses, as indicated by the altered stomatal density (SD; see Glossary, Box 1) in the *epf* mutants of grasses (Karavolias et al., 2023; Yin et al., 2017; Rathnasamy et al., 2023). Unlike *Arabidopsis*, however, stomatal development in grasses lacks amplifying divisions, suggesting that their EPF-regulated MAPK pathway may not target SPCH and functions differently compared with dicots (Rudall et al., 2017; Stebbins and Jain, 1960). For example, BdYODA is crucial for reinforcing SLGC/pavement cell fate post-asymmetric entry division; its mutants initially show normal epidermal divisions, but subsequent aberrant divisions in the larger daughter cells lead to clusters of stomata arranged in single rows (Abrash et al., 2018). Moreover, the ectopic expression of maize *SHORTROOT* in rice, which typically regulates cortical cell division, leads to the formation of supernumerary rows of stomatal cell files, which are more evenly spaced between veins (Schuler et al., 2018; Shaar-Moshe and Brady, 2023). This highlights the control of stomatal cell file patterning as an additional strategy in regulating stomatal number in grasses.

## Stomatal developmental responses to global warming-related stressors

### The effects of CO_2_ concentration

Throughout evolutionary history, the photosynthetic capacity of plants has been closely linked to their stomatal conductance (see Glossary, Box 1), a relationship that underscores the intricate balance between CO_2_ uptake and water loss (Woodward, 1987). Although elevated stomatal conductance enhances photosynthesis, it also entails risks, such as increased water loss and metabolic costs associated with constructing additional stomatal complexes (Brodribb and Feild, 2010). Consequently, stomatal adaptations to atmospheric CO_2_ levels have been proposed as a driving force in the evolution of land plants, with the early diversification of vascular plants and morphological advancements such as planate leaves coinciding with epochs of low atmospheric CO_2_ concentrations (Beerling et al., 2001; Haworth et al., 2011). As stomatal conductance scales directly with SD when CO_2_ is limited, it is unsurprising that plants can respond to atmospheric CO_2_ levels by adjusting stomatal production to maximize gas exchange capacity (Dow et al., 2014a). Empirical studies across 110 different plant species have revealed a generalized response towards varied CO_2_ concentrations, with an average SD reduction of 29% when CO_2_ levels doubled (Woodward and Kelly, 1995; Woodward et al., 2002). This observation is further supported by paleontological investigations; for example, analyses of fossil leaves from *Salix herbacea* over a glacial cycle spanning 140,000 years have revealed a negative correlation between SD and CO_2_ concentration (Beerling and Chaloner, 1993a).

Mutant analyses have suggested a role for fatty acid metabolism and abscisic acid (ABA) in the regulation of CO_2_-mediated suppression of stomatal development, although the underlying mechanisms remain unclear. In this context, the high carbon dioxide (*hic*) mutant in *Arabidopsis*, which is impaired in the biosynthesis of very-long-chain fatty acids and wax, exhibits fewer stomata than wild type under ambient CO_2_ levels but, surprisingly, exhibits an increased stomatal index (SI; see Glossary, Box 1) under elevated CO_2_ (Gray et al., 2000). Meanwhile, the *fad-4* mutant, which is defective in a chloroplastic fatty-acid desaturase, and the ABA-deficient double-mutant nine-cis-epoxycarotenoid dioxygenase 3 and 5 (*nced3 nced5*), do not show significant changes in stomatal production under elevated CO_2_ levels (Chater et al., 2015; Woodward et al., 2002). Interestingly, a systemic response to atmospheric CO_2_ concentration was demonstrated in a gas-tight cuvette experiment, where exposing the mature leaves of *Arabidopsis* to different CO_2_ concentrations significantly altered stomatal production in the young leaves (Lake et al., 2001). Although the specific mobile signals responsible for the long-distance CO_2_ signaling remain unknown, ABA, ethylene and jasmonate are implicated in the process, as mutants in these pathways show variable responses, highlighting the interplay of phytohormones in the CO_2_-triggered systemic suppression of stomatal development (Lake et al., 2001, 2002).

At the local tissue level, wild-type *Arabidopsis* modulates stomatal production via the EPF2-ERECTA signaling pathway in response to changes in CO_2_ levels, as elevated CO_2_ fails to reduce and even enhances stomatal production in both *epf2* and *erecta* mutants (Fig. 2) (Engineer et al., 2014). Further investigation employing apoplastic proteomics revealed that a protease, CO_2_ RESPONSE SECRETED PROTEASE (CRSP), promotes the cleavage and maturation of the EPF2 pro-peptide (Engineer et al., 2014). Notably, the transcript expressions of *CRSP* and *EPF2* significantly increase in response to high CO_2_ level, but this CO_2_-induced expression is absent in the β-carbonic anhydrase double mutant *ca1 ca4* (Engineer et al., 2014). CA1 and CA4 are known upstream regulators of CO_2_-triggered stomatal closure, and their mutations also lead to increased SI at elevated CO_2_ levels (Hu et al., 2010; Engineer et al., 2014). Although the molecular mechanisms by which the β-carbonic anhydrases regulate *EPF2* and *CRSP* expressions remain unclear, high atmospheric CO_2_ is believed to activate CA1 and CA4, and trigger EPF2 and CRSP production, leading to the activation of the downstream MAPK signaling pathway and suppression of stomatal development (Fig. 2).

### The effects of high temperature

Depending on the intensity and duration of the heat stress, the effect of high temperature on plants can be classified as either a high ambient temperature response or a heat shock response (Quint et al., 2016; Kotak et al., 2007; Zhou et al., 2022). The exact temperature range that induces these different responses is also species specific. For *Arabidopsis*, for example, prolonged exposure to 28-30°C leads to the high ambient temperature response, and short exposure at 37-40°C typically triggers the heat shock response (Zhou et al., 2022). At high ambient temperatures, plants undergo thermomorphogenesis, resulting in various developmental and morphological adaptations, including hypocotyl elongation, reduced leaf size and hyponasty (i.e. upward bending of plant parts such as petioles), all aimed at promoting cooling (Quint et al., 2016). Under extreme heat conditions, heat shock responses are activated through heat shock transcription factors (HSFs), which activate downstream heat response genes, and heat shock proteins (HSPs), which regulate proteostasis to promote thermotolerance and survival (Zhou et al., 2022).

Stomatal adaptations under high temperatures present a dual challenge: although plants require transpirational cooling for temperature regulation, they must also limit water loss because heat is often associated with drought (Radin et al., 1994). Hence, plants have evolved various stomatal responses towards high temperatures to ensure an optimal balance and their survival. For instance, the wild-type rice cultivar ‘IR64’ exhibits a 40% increase in SD when grown at high temperature (40°C), likely to promote evaporative cooling (Caine et al., 2019). However, *OsEPF1*-overexpressing plants, which were engineered to have significantly fewer stomata and limited ability to adjust stomatal production, cope with the high heat by significantly increasing their pore size rather than by modulating their SD (Caine et al., 2019). A similar compensatory response was observed in a hypomorphic mutant of *SPCH* in *Arabidopsis*, *spch-5*, which has extremely low SD. When grown at high ambient temperature for *Arabidopsis* (30°C), the mutant develops larger stomata and thinner leaves, likely to provide a cooling capacity similar to that of wild-type Col-0 plants (Perez-Bueno et al., 2022). These observations highlight a compensatory relationship between stomatal number and size for optimizing stomatal conductance in response to heat stress. High ambient temperature in *Arabidopsis* also promotes cell expansion and cell size increase in pavement and palisade cells, while reducing their numbers (Saini et al., 2022). This effect is mediated by the PHYTOCHROME-INTERACTING FACTOR 4 (PIF4) (see below) and TEOSINTE BRANCHED 1, CYCLOIDEA, AND PCF FAMILY 4 (TCP4) transcription factors, which regulate cell cycle progression. Whether the regulation of stomatal size by heat is also mediated by PIF4 and TCP4 remains to be examined.

Unlike CO_2_, the impact of high temperature on stomatal production varies significantly between plant species (Table 1). For instance, tobacco (*Nicotiana tabacum*), tomato (*Solanum lycopersicum*), perennial ryegrass (*Lolium perenne*), rose (*Rosa hybrida*) and soybean (*Glycine max*) show increased SD in response to higher temperatures (Hu et al., 2014; Pandey et al., 2007; Ferris et al., 1996; Jumrani et al., 2017; Nir et al., 2023 preprint). In contrast, common oak (*Quercus robur*), tea plants (*Camellia sinensis*) and subtropical forest tree (*Schima superba*) grown under warmer conditions produce fewer stomata (Beerling and Chaloner, 1993b; Wu et al., 2018; Li et al., 2023). Notably, variations in the high temperature response have also been observed within a species. Within *Brassica oleracea*, cabbage (*B. oleracea capitata*) exhibits increased SD at 32°C, whereas kale (*B. oleracea acephala*) shows no significant changes (Rodriguez et al., 2015). Even *Arabidopsis* accessions adapted to distinct climates demonstrate divergent responses to high temperatures: Col-0, originating from a cool, moist climate, displays decreased SI, whereas Bur-0 (from Ireland) and Kz-9 (from Kazakhstan) do not show the high temperature-induced suppression of stomatal development (Nir et al., 2023 preprint; Lau et al., 2018). This diversity in the stomatal response underscores the complex yet flexible nature of the stomatal adaptation to high temperature and the importance of habitat and breeding in shaping the developmental response.

**Table 1.**
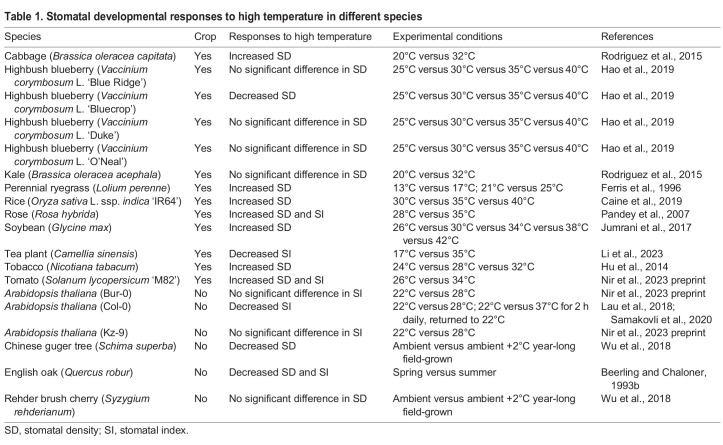
Stomatal developmental responses to high temperature in different species

Studies on the *Arabidopsis* Col-0 accession have examined how stomatal production is affected by both acute heat shock and high ambient temperature, unveiling distinct signaling mechanisms (Fig. 2). In response to daily, short-duration acute heat shock (37°C), stomatal production is suppressed (Samakovli et al., 2020). This is due to heat-activated heat shock protein 90s (HSP90s) interacting with YODA, which triggers MAPK signaling to ultimately suppress SPCH (Fig. 2). Chemical inhibition or genetic depletion of HSP90 disrupts this interaction, blocking the heat-induced MAPK cascade and subsequent SPCH degradation (Samakovli et al., 2020). In contrast, at elevated ambient temperature (28°C), a different regulatory mechanism controls SPCH levels in *Arabidopsis* by repressing the expression of the *SPCH* gene (Lau et al., 2018). Here, PIF4, a key transcription factor that promotes thermomorphogenic responses, accumulates and directly binds to the *SPCH* promoter through its E-box motifs, inhibiting its expression to repress stomatal development (Lau et al., 2018; Quint et al., 2016) (Fig. 2). Moreover, SPCH itself suppresses *PIF4* expression through direct binding, forming a negative-feedback loop that may limit *PIF4* suppression in earlier stomatal lineage cells under standard conditions (Lau et al., 2018). Interestingly, a recent study has demonstrated that tea plants (*Camellia sinensis*) grown at 35°C exhibit upregulated expression of *CsHSP90-1* and *CsHSP90-2*, alongside significantly suppressed expression of the *CsSPCHa* gene (Li et al., 2023). This suggests the potential conservation and activation of both the transcriptional and translational regulation of SPCH in response to heat stress in tea plants. Furthermore, in another study, gene-edited tomato plants (*Solanum lycopersicum*) that produce an N-terminal-truncated SPCH protein displayed a significantly altered stomatal response to high temperatures (Nir et al., 2023 preprint). Unlike wild-type tomatoes, in which high temperatures increase their SI, this mutant's SI was suppressed. Although its *in vivo* relevance remains unknown, fluorescent reporter analysis revealed that the truncated version of SlSPCH not only accumulates at a reduced level but that its nuclear localization is also significantly compromised at high temperatures. Overall, these studies point up the centrality of SPCH in the high temperature-mediated response of stomatal development.

The high temperature signaling pathways are intricately linked to the light signaling pathways (Jung et al., 2016; Legris et al., 2016; Delker et al., 2022). Light signaling regulates stomatal development in a light-dependent manner (Kang et al., 2009; Wang et al., 2021; Casson et al., 2009; Lee et al., 2017). In addition to their role in light signaling, photoreceptors, particularly phytochrome B (phyB), and other light signaling components also mediate high temperature response (Jung et al., 2016; Legris et al., 2016; Vu et al., 2019). For instance, elevated ambient temperatures convert phyB to its inactive form, which relieves the phosphorylation and inhibition of PIF4 and leads to thermomorphogenesis (Lorrain et al., 2008; Delker et al., 2022; Jung et al., 2016). Therefore, although dedicated studies are still lacking, high temperature-mediated stomatal formation may potentially act through other light signaling components as well. This highlights the multiple ways stomatal development may be controlled and the extensive crosstalk between abiotic pathways.

### The effect of drought and water availability

The fluctuating water availability resulting from climate change presents one of the most formidable challenges to plants for their growth and survival. As drought conditions become more prevalent, plants undergo physiological adaptations to conserve water and enhance water use efficiency (WUE). WUE, measured by a plant's ability to fix CO_2_ through photosynthesis while minimizing water loss via transpiration, is a key metric in assessing plant resilience to water stress (Condon et al., 2004). As stomata serve as crucial intermediaries that regulate both photosynthesis and transpiration, stomatal traits are a vital focus for enhancing WUE. Consequently, the effect of drought stands out as one of the most extensively studied abiotic stresses that impact stomatal development.

Studies investigating the effect of drought stress on stomatal production in diverse plant species have revealed that there are two major strategies adopted by plants (Table 2). The first strategy, understandably, involves a reduction in stomatal density. For instance, plant species including cotton (*Gossypium hirsutum*), oil palm (*Elaeis guineensis*), *Arabidopsis* (*Arabidopsis thaliana*) and balsam poplar (*Populus balsamifera*), exhibit a reduction in stomatal number when subjected to drought stress (Dubey et al., 2023; Song et al., 2023; Kumari et al., 2014; Hamanishi et al., 2012). This decrease in stomatal production is associated with an improvement in WUE, as it reduces water loss through transpiration, thereby enhancing drought tolerance. Numerous studies have therefore employed genetic engineering techniques, such as overexpressing *EPF1/2* or knocking out *STOMAGEN* in crops, to reduce stomatal number. This approach has been applied in rice (*Oryza sativa*), wheat (*Triticum aestivum*), barley (*Hordeum vulgare*), tomato (*Solanum lycopersicum*), poplar (*Populus tomentosa*), *Arabidopsis* (*Arabidopsis thaliana*) and grapevine (*Vitis vinifera*), resulting in plants with enhanced WUE and improved performance under drought conditions (Caine et al., 2019; Dunn et al., 2019; Hughes et al., 2017; Doheny-Adams et al., 2012; Wang et al., 2016; Jiang et al., 2019; Clemens et al., 2022; Karavolias et al., 2023; Yin et al., 2017). Notably, the reduction of SD in the transgenic plants often has minimal impact on their yield under well-watered conditions.

**Table 2.**
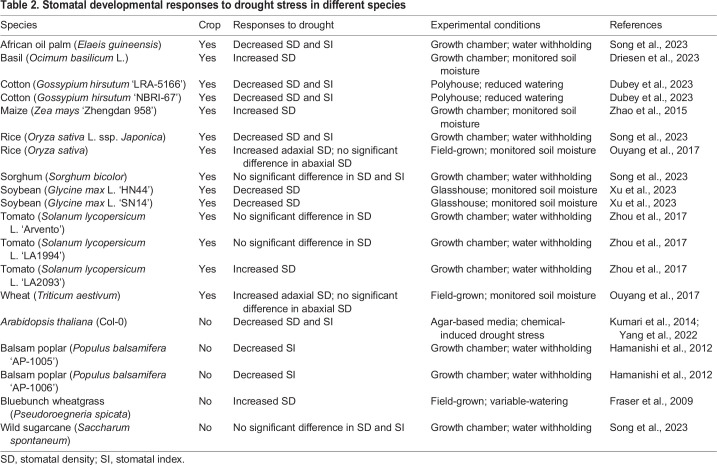
Stomatal developmental responses to drought stress in different species

The second strategy involves a surprising increase in SD but a reduction in the size of stomata. Whereas sugarcane (*Saccharum spontaneum*) and sorghum (*Sorghum bicolor*) show no changes in stomatal production under drought stress, species such as bluebunch wheatgrass (*Pseudoroegneria spicata*), rice (*Oryza sativa*), basil (*Ocimum basilicum*), maize (*Zea mays*), wheat (*Triticum aestivum*) and tomato (*Solanum lycopersicum*) increase their SD under similar conditions, accompanied by a notable reduction in stomatal size (Fraser et al., 2009; Driesen et al., 2023; Zhao et al., 2015; Ouyang et al., 2017; Zhou et al., 2017; Song et al., 2023). The combination of higher density and smaller-sized stomata may enable quicker responses to environmental cues and require less metabolic energy for aperture adjustment, potentially contributing to long-term WUE enhancement (Xu and Zhou, 2008; Hasanuzzaman et al., 2023; Caine et al., 2023). A recent study explored the effect of varying stomatal size and density on drought tolerance (Pitaloka et al., 2022). Through a mutagenized collection of purple rice cultivars, Pitaloka and colleagues characterized and classified 216 mutants into four distinct categories with different combinations of stomatal densities and sizes. Interestingly, mutants with both small-sized and low-density stomata exhibited superior WUE and biomass production under prolonged water-limiting conditions (Pitaloka et al., 2022). This highlights the promising role of reducing both stomatal density and size for enhancing drought stress tolerance.

Although these variations in stomatal adaptations to drought may underscore intrinsic differences among plant species, it is crucial to consider the methods used for applying drought stress and sampling stomatal traits that may contribute to the observed differences. For instance, in the perennial grass *Leymus chinensis*, moderate water deficits have been shown to enhance stomatal numbers, whereas more severe deficits are associated with a reduction (Xu and Zhou, 2008). Additionally, although SD offers insights into the effects of drought stress on stomatal development, it may not fully encapsulate the complexity, as drought stress may lead to a significant decrease in leaf area (Xu and Zhou, 2008; Kouwenberg et al., 2007; Granier and Tardieu, 1999; Aguirrezabal et al., 2006). An inverse relationship between leaf size and stomatal density has been frequently observed across plant species, which presumably ensures adequate stomata in smaller leaves to maintain CO_2_ flux and photosynthetic capacity (Hasanuzzaman et al., 2023; Franks and Farquhar, 2007; Xu and Zhou, 2008). This stresses the importance of employing appropriate drought treatment regime(s) specific to the investigation and making comprehensive measurements of leaf and stomatal traits when assessing how plants respond to drought.

Research on how drought and osmotic stress inhibit stomatal development in *Arabidopsis* has highlighted the crucial role of SPCH protein regulation (Fig. 2). Although drought and osmotic stress originate from different conditions (lack of water versus high solute concentration), they can trigger similar signaling pathways, which are broadly divided into ABA-dependent and -independent pathways (Yoshida et al., 2014). Under mannitol-induced osmotic stress, the formation of both stomatal precursor cells and other epidermal cells decreases, likely owing to reduced accumulation of SPCH protein (Kumari et al., 2014). Chemical inhibition of the MAPK signaling pathway or the use of a MAPK-insensitive SPCH variant (S1-4A) reduces the severity of the osmotic stress-induced SPCH suppression, suggesting that osmotic stress activates the MAPK signaling pathway to inhibit stomatal production (Kumari et al., 2014). However, ABA, the key stress phytohormone that accumulates during drought and osmotic stress (Yoshida et al., 2014), also has a prominent role in suppressing stomatal initiation and development, as evidenced by the increased stomatal number and prolonged *SPCH* expression observed in the ABA-deficient mutant *aba2-2* (Tanaka et al., 2013). Using mutants with altered ABA levels and signaling, a recent study further showed that ABA plays a role in the proper patterning of stomata in *Arabidopsis* (Mohamed et al., 2023). Regarding the signaling mechanism, a recent study from our group demonstrated that the core signaling kinase of the ABA signaling pathway, SnRK2s, directly phosphorylates specific residues of SPCH to promote SPCH degradation (Yang et al., 2022). Interestingly, mutations in the SnRK2-targeted sites render SPCH largely unresponsive to ABA-triggered degradation, whereas MAPK-targeted site mutations retain sensitivity to ABA-induced degradation (Yang et al., 2022). This underscores the ability of SPCH to discern and integrate different signaling pathways through distinct phosphocodes to regulate stomatal development.

## Outstanding questions

Why and how the distinct adaptation strategies in stomatal developmental responses emerged remain poorly characterized. One such factor could be related to the photosynthetic process that a plant adopts. For example, C_4_ plants possess a carbon-concentrating mechanism that enhances the efficiency of RuBisCO (ribulose-1,5-bisphosphate carboxylase/oxygenase) and reduces photorespiration, which is absent in C_3_ plants (Sage, 2004). In the evolution from C_3_ to C_4_ within the *Flaveria* genus, this transition is marked by a reduction in SD and conductance – adaptations that significantly enhance WUE and allow C_4_ plants to maintain higher photosynthetic efficiency and water conservation compared with their C_3_ counterparts (Zhao et al., 2022). These adaptations may contribute to the differing sensitivity of stomatal production to environmental stressors. This notion is further supported by an independent study examining the effects of drought on several C_3_ and C_4_ plants, which suggests that drought significantly suppresses stomatal development in C_3_ plants more than in C_4_ plants (Song et al., 2023). Interestingly, even within the same species, drought-tolerant or drought-susceptible variants show divergent responses in stomatal production when subjected to drought (Song et al., 2023; Nguyen et al., 2024). Thus, these studies also highlight the significant role of local adaptation in shaping the stomatal response. Additionally, evidence suggests that the redox status of the photosynthetic electron transport chain can regulate stomatal development through the MAPK signaling pathway (Zoulias et al., 2021). The photosynthetic electron transport chain redox state affects reactive oxygen species (ROS) production, with reduced photorespiration in C_4_ plants leading to lower ROS levels (Dellero et al., 2016; Sonmez et al., 2022). Consequently, C_4_ plants modulate ROS-dependent signaling pathways differently than C_3_ plants, and show distinct ROS profiles and dynamics (Sonmez et al., 2022). Given that ROS play a dual role in damaging cellular components and signaling stress responses, the lower ROS levels in C_4_ plants may lead to different regulatory mechanisms for stomatal development, potentially offering greater resilience under stress conditions (Sonmez et al., 2022). This differential redox regulation could help explain how plants with different photosynthetic systems respond to environmental changes in stomatal development.

The combined effects of environmental stressors on stomatal development, anticipated to converge in future climate scenarios, also remain unclear. Woodward and colleagues reported that drought stress enhances the reduction in SD induced by elevated CO_2_, whereas Doheny-Adams et al. did not observe such enhanced sensitivity, suggesting that differences in drought severity and duration might account for these discrepancies (Woodward et al., 2002; Doheny-Adams et al., 2012). Although it is widely hypothesized that elevated CO_2_ helps plants conserve water under mild drought conditions by reducing stomatal conductance, it has been suggested that these benefits may diminish during severe drought (De Kauwe et al., 2021). In such scenarios, despite initial water conservation efforts, plants might still undergo increased water loss due to transpiration through their cuticles and minimal conductance from incompletely closed stomata (Liang et al., 2023). A recent meta-analysis of 616 published papers revealed that, although the combined effects of stressors such as elevated CO_2_, temperature and moisture on stomatal conductance are generally additive, they often become antagonistic to one another as their effect sizes increase (Liang et al., 2023). This shift from additive to antagonistic responses highlights the complex nature of environmental interactions affecting stomatal function, and poses significant challenges in accurately predicting how combined environmental stresses may impact stomatal development. This underscores the necessity for a refined approach to studying environmental impacts on plant physiology, emphasizing the need for comprehensive investigations that consider multiple interacting stressors.

Despite advancements in our understanding of stomatal initiation and division regulation, the control of stomatal size remains elusive. Notably, a high-throughput study on 330 *Arabidopsis* accessions indicated a stronger association between stomatal size and WUE compared with SD, emphasizing the need to elucidate the mechanisms governing stomatal size (Dittberner et al., 2018). Expanding upon earlier hypotheses on genome size/cell size relationship, a comprehensive study across 101 angiosperm species demonstrated a positive correlation between genome size and stomatal size, suggesting a co-evolutionary pattern, albeit with variations observed across different growth forms, such as trees, shrubs and herbs (Beaulieu et al., 2008). In *Arabidopsis* leaf epidermis, endoreduplication events in trichomes and pavement cells facilitate the development of larger cell sizes (Melaragno et al., 1993). However, given that guard cell nuclei typically do not undergo endoreduplication, it remains unclear how some mutants develop altered stomatal sizes, and further validation is required to determine whether these changes are a direct consequence of increased DNA content in GCs (Liang et al., 2010; Larson-Rabin et al., 2009; Wu et al., 2022; Yu et al., 2008; Guo et al., 2019; Des Marais et al., 2014).

Although the genetic control of stomatal size is unclear, a strong negative relationship between stomatal size and SD has been frequently observed (Franks et al., 2009; Li et al., 2020; Dittberner et al., 2018; Doheny-Adams et al., 2012; Nunes et al., 2022; Sun et al., 2014). Theories suggest this negative relationship might be due to geometric constraints and competition for space allocation between stomata and other non-stomatal cells on the leaf surface, such as trichomes and glands (Franks and Beerling, 2009; de Boer et al., 2016; Zhang et al., 2021). Hence, simultaneous manipulation of both stomatal size and density might be difficult to achieve. Intriguingly, a rare case in *Brachypodium* revealed that a mutation in a hair-cell specific peroxidase decreases prickle hair cell size, which indirectly leads to larger stomata without changes in SD, resulting in higher stomatal conductance (Nunes et al., 2023). This suggests that the negative linkage between stomatal size and SD may be influenced indirectly through manipulation of other epidermal lineage cells, necessitating more research to understand this complex developmental interplay. Supporting this idea, genetic studies in *Arabidopsis* and *Brachypodium* have provided some clues about the regulatory relationship between stomatal and trichome lineages. For example, overexpression of *TMM* in *Arabidopsis* results in clustering of stomata on the epidermis as well as aberrant trichome branching and significant suppression of trichome production (Nadeau and Sack, 2002; Yang and Sack, 1995; Yan et al., 2014). Similarly, the *bdyoda* loss-of-function mutant displays significant clusters and increased densities of various epidermal cell types, including stomata, hair and silica cells (Abrash et al., 2018). Understanding these inter-lineage communications could be an invaluable tool for engineering and optimizing epidermal cell traits and maximizing crop productivity.

## Concluding remarks

In this Review on the effects of climate change on stomatal development, we have consolidated key findings on the responses of diverse plant species to CO_2_, high temperature and drought, and the signaling pathways involved in the model plant *Arabidopsis*. Notably, although high CO_2_ concentrations generally lead to suppression of stomatal formation, the stomatal responses to high temperatures and drought exhibit strong species-specific variations, suggesting that plant adaptation strategies may differ significantly. The master transcription factor SPCH and members of the EPFL family also serve as key nodes in connecting environmental signaling pathways to stomatal development.

Building on the fundamental knowledge of stomatal formation, ongoing efforts are aimed at engineering stomatal traits for climate resilience. Members of the *EPFL* family are the main focus of these efforts because *EPF1* and *ERL2*, together with other regulatory genes, consistently emerge as key differentiators in local adaptation, as shown by historical stomatal development trends (Lang et al., 2024). The importance of EPFL-mediated signaling during evolution is also highlighted in the C_3_-to-C_4_ transition of *Flaveria*, whereby modulation of *STOMAGEN* expression levels may have resulted in decreased SD and higher WUE in the more efficient C_4_ species (Zhao et al., 2022). In addition, the *EPFL* family member *STOMAGEN* is light-responsive in *Arabidopsis*, enabling it to perceive environmental changes and regulate stomatal abundance (Wang et al., 2021; Hronkova et al., 2015) (Fig. 2). Thus, the tunability of EPF signaling in modulating stomatal formation makes this pathway an attractive target, and numerous studies have employed CRISPR-based genome-editing approaches to mutate *EPFL* genes, yielding crop plants with altered stomatal numbers to enhance their adaptation to diverse environmental conditions (Karavolias et al., 2023; Clemens et al., 2022; Rathnasamy et al., 2023; Yin et al., 2017). The exogenous application of EPFL peptides presents another avenue for SD manipulation, an approach that has been demonstrated in *Arabidopsis* (Sugano et al., 2010; Lee et al., 2012; Kondo et al., 2010). However, further research is required to enable economical large-scale production of these cysteine-rich peptides and to optimize application strategies.

Advancements in high-throughput phenotyping tools, combined with computational analysis, hold great promise for enabling rapid measurements of stomatal traits. Optical topometry, which accelerates the acquisition of epidermal patterning data through rapid image capture, has been successfully utilized to assess changes in SD in *Arabidopsis* in response to elevated CO_2_ levels (Haus et al., 2015, 2018). Furthermore, the adoption of deep-learning algorithms enhances the capability for large-scale pattern recognition and data analysis, integrating advanced imaging technologies to generate vast datasets on various stomatal traits (Bheemanahalli et al., 2021; Pignon et al., 2021; Ferguson et al., 2021). Combined with genome-wide association studies, this approach enables detailed characterization of stomatal traits, effectively linking genetic variations to stomatal phenotypes (Bheemanahalli et al., 2021; Xie et al., 2021; Dittberner et al., 2018).

Insights from fundamental research, combined with technical advancements, offer a framework for understanding how stomata develop and respond to changing environmental conditions. Leveraging this knowledge, it will be possible to develop crop varieties that are more resilient to extreme climate conditions and more water efficient. Additionally, this understanding could improve climate models by integrating plant physiological responses to environmental changes. These advancements are crucial for meeting the global food demands and ensuring sustainable agricultural productivity in the face of climate change.
